# The diagnostic value of serum C-reactive protein/albumin and homocysteine/high-density lipoprotein-cholesterol in coronary microvascular angina pectoris

**DOI:** 10.1590/1806-9282.20241772

**Published:** 2025-06-16

**Authors:** Wenxin Zhao, Xianfeng Zhao, Zhongyan Li

**Affiliations:** 1 Dalian Medical University, The Second Affiliated Hospital, Department of Cardiology – Dalian, China.; 2 Fuwai Central China Cardiovascular Hospital, Department of Cardiology – Zhengzhou, China.

**Keywords:** C-reactive protein, Albumin, Homocysteine, High-density lipoproteins, Microvascular angina

## Abstract

**OBJECTIVE:**

The aim of this study was to investigate the correlation between the serum C-reactive protein/albumin ratio and homocysteine/high-density lipoprotein-cholesterol ratio with microvascular angina and assess their predictive value.

**METHODS:**

A total of 70 patients diagnosed with microvascular angina comprised the observation group, while 68 patients with normal or minimal (<50%) coronary stenosis and normal coronary blood flow formed the control group. Serum C-reactive protein, albumin, homocysteine, high-density lipoprotein-cholesterol, and other indexes were measured, and C-reactive protein/albumin and homocysteine/high-density lipoprotein-cholesterol ratios were calculated. Logistic regression, Pearson's correlation, and receiver operating characteristic analyses were conducted to identify independent risk factors for microvascular angina.

**RESULTS:**

Significant differences in C-reactive protein, C-reactive protein/albumin, homocysteine, and homocysteine/high-density lipoprotein-cholesterol levels were found between the microvascular angina and control groups (p<0.05). Multivariate logistic regression analysis showed that C-reactive protein, C-reactive protein/albumin, homocysteine, and homocysteine/high-density lipoprotein-cholesterol were independent risk factors for microvascular angina, with the risk increasing alongside elevated C-reactive protein/albumin and homocysteine/high-density lipoprotein-cholesterol levels. The receiver operating characteristic analysis demonstrated that C-reactive protein/albumin, homocysteine/high-density lipoprotein-cholesterol, and their combined use were predictive of microvascular angina, with the combined area under the curve value exceeding that of individual markers.

**CONCLUSIONS:**

Elevated C-reactive protein/albumin and homocysteine/high-density lipoprotein-cholesterol offer predictive value for microvascular angina diagnosis, with the combination providing superior diagnostic accuracy over individual indicators, supporting early microvascular angina identification.

## INTRODUCTION

Microvascular angina (MVA) is a clinical syndrome characterized by acute and chronic myocardial ischemia resulting from structural and functional abnormalities in coronary arterioles, small arteries, and capillaries^1-3^. This condition may be caused by atherosclerotic factors, such as endothelial dysfunction, and non-atherosclerotic factors, including microvascular spasm^4^. Recent studies have shown that patients with MVA face a significantly higher risk of major cardiovascular events, such as heart failure and acute coronary syndrome, highlighting the need for timely identification and intervention. Currently, diagnostic methods for MVA include coronary angiography, intra-coronary Doppler flow conductance wire, and other invasive procedures, as well as non-invasive techniques such as cardiac magnetic resonance imaging, positron emission tomography, and single-photon emission computed tomography (CT)^5-8^. However, the widespread use of these technologies is limited due to their technical complexity and high costs. Consequently, a simple, non-invasive, and reliable serological diagnostic method for MVA is urgently needed.

MVA is a clinical syndrome characterized by acute and chronic myocardial ischemia caused by structural and functional abnormalities in coronary microvessels, including arterioles, small arteries, and capillaries^9^. C-reactive protein (CRP), a marker of acute-phase inflammation, is positively correlated with inflammation levels and plays a role in diagnosing and predicting the prognosis of coronary heart disease (CHD). Inflammatory processes often reduce albumin (ALB) levels, a negative acute-phase protein, which is inversely associated with the occurrence of cardiovascular diseases. Therefore, the CRP-to-ALB ratio (CAR) may more accurately reflect the inflammatory status associated with CHD. Patients with MVA are at an increased risk of major cardiovascular events, such as heart failure and acute coronary syndrome, underscoring the critical need for timely identification and intervention. Current diagnostic approaches, including invasive procedures such as coronary angiography and intra-coronary Doppler flow measurements, and advanced non-invasive imaging modalities like cardiac magnetic resonance imaging and positron emission tomography are limited by their technical complexity, invasiveness, and high costs. These limitations highlight the urgent need for simple, non-invasive, and cost-effective serological diagnostic methods for MVA.

Studies have also shown^10^ that elevated HCY levels are strongly associated with atherosclerosis, promoting its progression by influencing oxidative stress, vascular endothelial dysfunction, immune-inflammatory responses, and vascular smooth muscle cell proliferation. HDL-C is involved in reverse cholesterol transport and alleviates atherosclerosis through anti-inflammatory, antioxidant, and endothelial-protective effects. Thus, the homocysteine/high-density lipoprotein-cholesterol ratio (HCY/HDL-C ratio) can partially indicate endothelial damage due to oxidative stress and lipid metabolism disorders. These ratios can be measured through blood tests, making them cost-effective and time-efficient.

Despite their potential, research on the diagnostic utility of CAR and HCY/HDL-C in MVA remains limited. This study aimed to address this gap by evaluating the associations between these ratios and MVA, and by assessing their diagnostic value using serum levels of CRP, ALB, HCY, and HDL-C in patients with MVA. By combining these biomarkers, this study sought to advance non-invasive diagnostic strategies for MVA, offering practical advantages over existing methods. This novel approach has the potential to enhance early diagnosis and improve the clinical management of MVA, ultimately addressing a critical unmet need in cardiovascular care.

## METHODS

### General information

This study included 70 patients diagnosed with MVA who were admitted to the Second Affiliated Hospital of Dalian Medical University between September 2022 and December 2023. The observation group consisted of 33 females and 37 males, with an average age of 58.78±9.27 years. Additionally, 68 patients with normal or mild coronary artery stenosis <50%, based on coronary angiography and negative exercise treadmill tests, selected randomly constituted the control group. This group comprised 33 females and 35 males, with an average age of 60.62±10.11 years. This study was approved by the Ethics Committee of the Second Affiliated Hospital of Dalian Medical University (DMU-22-031). Signed written informed consents were obtained from the patients and/or guardians.

The diagnostic criteria for MVA were based on the 2018 International Coronary Vasomotion Disorders International Study Group and COVADIS guidelines. The diagnostic criteria include: (1) symptoms of myocardial ischemia such as angina pectoris or shortness of breath; (2) coronary angiography showing normal or narrow coronary arteries <50%; (3) positive electrocardiogram exercise test or exercise treadmill test; and (4) slow blood flow in the coronary arteries: at least one coronary artery with corrected thrombolysis in myocardial infarction (TIMI) blood flow frame number >27 frames (images collected at a rate of 30 frames per second). The inclusion criteria comprised: (1) patients aged 37–85 years admitted to the hospital for chest pain for coronary angiography or coronary CT examination; (2) clear mental status, normal communication ability, and absence of serious neuropsychiatric diseases; and (3) meeting the diagnosis of MVA.

The exclusion criteria for MVA encompassed: (1) patients with a history of acute myocardial infarction, coronary stent implantation, coronary artery bypass surgery, or other revascularization procedures; (2) hypertensive heart disease, rheumatic heart disease, cardiomyopathy, heart failure, aortic dissection, or other organic heart diseases; (3) serious liver and kidney dysfunction; (4) suspected non-cardiogenic chest pain due to pleural or intercostal neuralgia; (5) uncontrolled, symptomatic or hemodynamic arrhythmias; (6) the presence of active inflammation (immune-related diseases, respiratory infections, etc.); and (7) diagnosis of malignant tumors.

### Methods

#### Data collection

Clinical data, including age, sex, body mass index (BMI), and a history of hypertension, diabetes, and smoking, were collected at admission. Early morning fasting vein (primary vein or median vein) blood of all patients was collected after admission to detect liver function, including ALB, alanine aminotransferase (ALT), and aspartate aminotransferase (AST). HDL-C, CRP, HCY, and serum creatinine (sCr) were detected by an automatic biochemical analyzer (Beckman, USA), and CAR and HCY/HDL-C were calculated. The left ventricular ejection fraction (LVEF) of all patients was measured by Siemens color Doppler ultrasound (G603, probe frequency 3–4 MHz).

#### Grouping of patients

Patients were categorized based on coronary angiography results and exercise testing outcomes. According to the results of coronary angiography, patients with no obvious abnormality or stenosis <50% and with positive results of exercise plate test or 12-lead Holter electrocardiogram (ST-segment downslope or horizontal depression ≥0.1 mv) were selected as the observation group (n=70). Patients with <50% vascular stenosis or no significant abnormality and with negative results of treadmill exercise test or Holter electrocardiogram constituted the control group (n=68).

### Statistical analysis

Statistical analysis was conducted using SPSS 26.0 and GraphPad Prism 9.5. The Shapiro-Wilk test assessed data normality. Normally distributed data were expressed as mean±standard deviation and compared via t-tests, while non-normal data were expressed as median (P25, P75) and analyzed using the Mann-Whitney U test. Categorical data were presented as counts (percentages) and analyzed using the chi-square test. Multivariate logistic regression analysis identified independent variables, and receiver operating characteristic (ROC) curves determined the predictive value of CAR and HCY/HDL-C for MVA, with p<0.05 indicating statistical significance.

### Determination of cutoff values

ROC curve analysis was used to determine the optimal cutoff values for CAR and HCY/HDL-C in diagnosing MVA. Sensitivity, specificity, positive predictive value (PPV), and negative predictive value (NPV) were calculated for different thresholds. The Youden Index (sensitivity+specificity-1) was used to identify the threshold that maximized the balance between sensitivity and specificity.

### Significance testing

Area under the curve (AUC) value comparisons between CAR and HCY/HDL-C were conducted to evaluate which ratio had superior predictive performance. A p<0.05 was considered ­statistically significant.

## RESULTS

### General data comparison

There were no statistically significant differences in terms of gender, age, BMI, and a history of hypertension, diabetes, and smoking between the two groups (p>0.05; Table 1).

**Table 1:** Comparison of clinical data and laboratory indicators between the two groups (n [%], x±s, M [P25, P75]).

Clinical indices	MVA group (n=64)	Non-MVA group (n=68)	t/z/χ^2^	p
Age (x±s)	57.66±10.02	60.62±10.11	-1.727	0.086
Gender (%)	37 (52.9)	35 (51.5)	0.027	0.871
Smoking status (%)	34 (48.6)	24 (35.3)	2.496	0.114
Hypertension (%)	37 (52.9)	39 (57.4)	0.282	0.596
BMI (x±s)	25.16±2.91	25.25±2.96	-0.714	0.862
AST (U/L, M [Q1, Q3])	22.56 (16, 28)	22.74 (18, 26)	-0.781	0.435
ALT (U/L, M [Q1, Q3])	23.89 (15, 26.25)	23.03 (16, 25.75)	-0.200	0.841
sCr (mg/dL, M [Q1, Q3])	67.36 (55.32, 77.97)	66.92 (51.94, 83.31)	-0.635	0.526
LVEF (%, M [Q1, Q3])	60 (58, 62)	60 (61, 59)	-1.308	0.191
LDL-C (mmol/L, M [Q1, Q3])	2.94 (2.28, 3.42)	2.75 (2.24, 3.23)	-0.488	0.626
HDL-C (mmol/L,M [Q1, Q3])	1.31 (1.04, 1.60)	1.22 (1.11, 1.32)	-0.914	0.361
HCY (μmol/L, x±s)	16.70±4.80	13.64±2.36	4.520	<0.001
CRP (mg/dL, x±s)	5.13±1.69	3.57±0.87	6.733	<0.001
ALB (g/L, x±s)	41.74±3.13	42.18±3.42	-0.765	0.446
CAR*100 (x±s)	12.37±4.25	8.54±2.24	6.540	<0.001
HCY/HDL-C (x±s)	13.59±5.36	11.15±2.83	3.336	<0.001

BMI: body mass index; LDL-C: low-density lipoprotein-cholesterol; HDL-C: high-density lipoprotein-cholesterol; AST: aspartate aminotransferase; ALT: alanine aminotransferase; sCr: serum creatinine; LVEF: left ventricular ejection fraction; CRP: C-reactive protein; ALB: albumin; CAR: C-reactive protein/albumin ratio; HCY: homocysteine; HCY/HDL-C: homocysteine/high-density lipoprotein-cholesterol; MVA: microvascular angina. T-value: The t test is used to compare the sample mean when the normal distribution is met. Z-value: Used to assess deviation from the population mean in non-normal distributions. χ^2^ value: Used in categorical data analysis for independence tests, goodness of fit, or variance comparisons.

### Comparison of laboratory data

There were no statistically significant differences in AST, ALT, ALB, sCr, LDL-C, HDL-C, and LVEF between the two groups (p>0.05). There were statistically significant differences in CRP, CAR, HCY, and HDL-C between the two groups, and the CRP, CAR, HCY, and HCY/HDL-C levels in the observation group were higher than those in the control group (p<0.001; Table 1).

### Comparison of serum C-reactive protein/albumin and homocysteine/high-density lipoprotein-cholesterol levels between the observation group and control group

The serum CAR level in the observation group (12.37±4.25) was statistically significantly higher than that in the control group (8.54±2.24) by group t-test (p<0.001). Similarly, serum HCY/HDL-C in the two groups was statistically significantly higher in the observation group (13.59±5.36) than that in the control group (11.15±2.83) by group t-test (p<0.001). The CAR was calculated as the ratio of serum C-reactive protein (CRP, measured in mg/dL) to albumin (ALB, measured in g/dL), while HCY/HDL-C was calculated as the ratio of serum homocysteine (HCY, measured in μmol/L) to high-density lipoprotein-cholesterol (HDL-C, measured in mmol/L). Standardized units were used for all measurements to ensure consistency and comparability across the study groups. These ratios were computed using data obtained from fasting blood samples analyzed with an automated biochemical analyzer (Table 1).

### Logistic regression analysis of microvascular angina risk factors

Multivariate logistic regression analysis was used to evaluate the association between statistically significant variables such as CRP, CAR, HCY, and HCY/HDL-C as independent ­variables and MVA as a dependent variable (assigned 0=control group, 1=MVA group [observation group]). The results showed that CRP, CAR, HCY, and HCY/HDL-C were independent risk factors for MVA, and the risk of MVA increased with the increase of serum CAR and HCY/HDL-C levels (Table 2).

**Table 2:** Multivariate logistic regression analysis of microvascular angina risk factors.

Variables	β	OR	95%CI	p
CRP	0.056	1.057	0.640–1.745	<0.001
HCY	0.199	1.220	1.099–1.355	0.003
CAR*100	0.752	2.122	0.609–3.390	<0.001
HCY/HDL-C	0.522	1.730	1.035–2.235	<0.001

OR: odds ratio; CI: confidence interval; CRP: C-reactive protein; CAR: C-reactive protein/albumin ratio; HCY: homocysteine; HCY/HDL-C: homocysteine/high-density lipoprotein-cholesterol. β value often represents coefficients in regression analysis and can be used to assess the extent to which the independent variable influences the dependent variable, as well as the predictive accuracy of the dependent variable.

### Serum C-reactive protein/albumin, homocysteine/high-density lipoprotein-cholesterol, and their combination for microvascular angina prediction analysis

**Figure 1:**
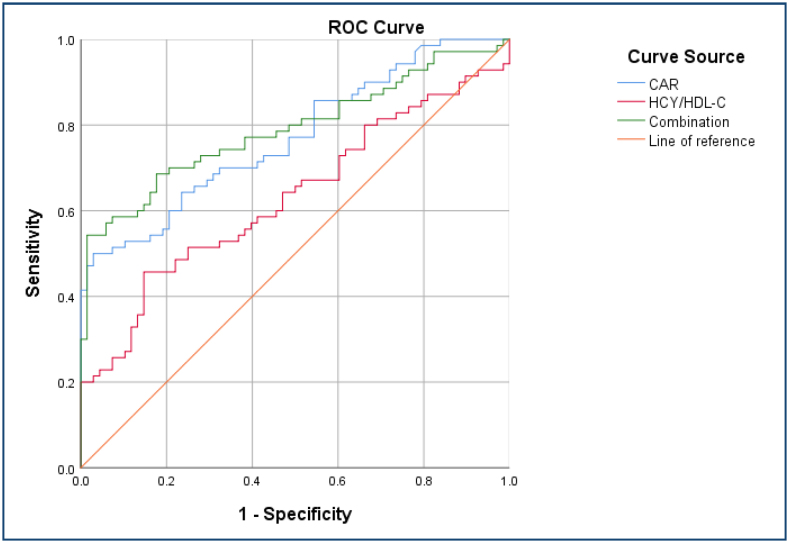
Serum C-reactive protein/albumin, homocysteine/high-density lipoprotein-cholesterol, and their combination in the diagnosis of microvascular angina pectoris (receiver operating characteristic curve). ROC: receiver operating characteristic; CAR: C-reactive protein/albumin ratio; HCY/HDL-C: homocysteine/high-density lipoprotein-cholesterol.

Serum CAR and HCY/HDL-C were used as clinical indicators to evaluate MVA, and their predictive value was assessed using ROC curve analysis. The AUC value for serum CAR and HCY/HDL-C in diagnosing MVA patients was 0.771 and 0.531, respectively. The optimal cutoff values were determined to be 0.12 for CAR, which showed a sensitivity of 68.4% and a specificity of 97.1%, and 13.36 for HCY/HDL-C, with a sensitivity of 45.7% and a specificity of 85.3%. When combined, the two indicators provided an AUC value of 0.790, enhancing the sensitivity to 74.3% and the specificity to 98.5%. Statistical analysis revealed significant differences in the AUC values between CAR and HCY/HDL-C, CAR alone versus the combined indicators, and HCY/HDL-C alone versus the ­combined indicators (p<0.05), underscoring the superior ­diagnostic accuracy of using both markers in tandem (Figure 1).

## DISCUSSION

Studies have shown that inflammation is involved in the occurrence, development, and deterioration of CHD, which eventually leads to vascular inflammation, plaque rupture, and thrombosis, threatening the life of patients. MVA is a type of CHD, and its diagnostic criteria are signs and symptoms of myocardial ischemia, decreased coronary blood flow reserve or microvessel spasm, and documented myocardial ischemia, which are not caused by obstructive CHD but by functional or structural abnormalities of the coronary microcirculation site. In order to diagnose MVA, patients need to undergo multiple coronary angiography, which is a heavy medical burden for patients and seriously affects their quality of life. According to the National Heart, Lung and Blood Institute, MVA patients have a poor prognosis, and MVA is also a reliable independent predictor of adverse cardiovascular events.

MVA is the result of multiple factors and mechanisms, including vascular endothelial dysfunction, inflammatory response, hemorrheological abnormalities, and cardiac autonomic nervous dysfunction, which are the causes of abnormal coronary microcirculation. A recent report has shown that^11^ vascular endothelial function injury is one of the main causes of coronary microcirculation diseases, in which inflammation plays a key role, such as CRP and HCY, and it can promote the activation and functional abnormalities of vascular endothelial cells and further lead to abnormal microcirculation.

Previous evidence has shown that^12^ CRP can damage the activation of the complement system and endothelial ­progenitor cells, inhibit fibrinolysis and increase collagen degradation, and may participate in the uptake of LDL-C by macrophages to transform it into foam cells, which directly harm the vasomotor function of human endothelium-derived blood vessels and have a pro-inflammatory effect on vascular endothelial cells. It plays an important role in the occurrence and development of CHD. Evidence also showed that^13^ decreased serum albumin levels correlate with the chronic nature of the disease and represent an inflammatory state. In addition, decreased albumin levels were found to be associated with increased blood viscosity, impaired endothelial function, increased platelet activation and aggregation, and increased synthesis of important mediators of platelet-derived coronary artery stenosis. CRP and ALB are positive and negative factors, respectively, in inflammatory response, and hence CAR has an amplification effect compared with a single indicator and can reflect the activity of inflammation more accurately. Karabağ et al. found that compared with CRP and ALB, CAR had more advantages in predicting coronary artery disease and was an independent predictor in the group of moderate- and high-risk coronary artery diseases^14^.

HCY can cause atherosclerosis and lead to CHD through the following five ways: (a) damaging and dysfunctioning vascular endothelial cells; (b) causing abnormal blood ­lipids; (c) stimulating vascular smooth muscle cell proliferation; (d) enhancing coagulation function and inducing thrombosis; and (e) promoting the expression of inflammatory ­factors^15^. In addition, hyperhomocysteinemia (hyperHcy) can also increase the expression of CD36, the scavenger receptor of oxidized-LDL (ox-LDL), in macrophages, resulting in the formation of foam cells. HDL-C, as an independent protective factor in CHD, can directly antagonize the effects of ox-LDL on endothelial cells and smooth muscle cells, and its antioxidant capacity is mainly derived from proteins related to HDL-C, such as PON1 (paraoxonase-1, human paraoxonase-1) and ApoA-1 (apolipoprotein A-1). PON1 protein can prevent the oxidative modification of LDL-C and the subsequent formation of atherosclerotic lesions. HCY can inhibit the activity of PON1, make HDL-C lose its cardiovascular protective effects such as antioxidant and anti-toxicity, and play a promoting role in the formation of atherosclerosis^16^. In summary, plasma HCY level in patients with CHD is negatively correlated with HDL-C, and hyperhcy can promote the clearance of HDL-C. Therefore, HCY/HDL-C can reflect the disorder of lipid metabolism and vascular endothelial damage in patients with CHD to a certain extent.

The results of this study showed that serum CAR (12.37±4.25 vs. 8.54±2.24, p<0.001) and HCY/HDL-C (13.59±5.36 vs. 11.15±2.83, p<0.001) levels in the MVA group were significantly higher than those in the control group, and the ­differences were statistically significant. Multivariate logistic regression analysis showed that serum CAR (OR 2.122, 95%CI 0.609–3.390, p<0.001) and HCY/HDL-C (OR 1.730, 95%CI 1.035–2.235, p<0.001) levels were independent risk factors for MVA. In addition, ROC curve and AUC analyses showed that serum CAR combined with HCY/HDL-C had good predictive value for MVA diagnosis and yielded good PPV for MVA diagnosis (AUC=0.790), with a sensitivity of 74.3% and a specificity of 98.5%.

The use of CAR and HCY/HDL-C in routine clinical practice offers several advantages. These biomarkers are derived from standard blood tests that are already widely available, making them cost-effective and accessible, particularly in resource-limited settings. Unlike imaging-based methods, which require specialized equipment and expertise, CAR and HCY/HDL-C can be measured with existing laboratory infrastructure, significantly reducing the diagnostic burden on healthcare systems. Incorporating these markers into clinical workflows could provide an efficient first-line screening tool for patients ­presenting with the symptoms of myocardial ischemia but without obstructive coronary disease. For example, patients with elevated CAR and HCY/HDL-C levels could be prioritized for advanced diagnostic procedures, such as coronary angiography or functional imaging. Additionally, their dynamic changes over time could be monitored to assess disease progression or treatment response, which may further enhance their clinical utility.

In summary, the combination of CAR and HCY/HDL-C appears to be critical in the occurrence and development of MVA. However, the limitations of this study should not be ignored: First, this study was a single-center, case–control study, and all subjects were from the same hospital during the same period, so there may be selection bias. Second, the sample size of this study is small and the geographical scope is limited, which may limit the persuasiveness of the study conclusions to a certain extent. Therefore, further multicentric and large-sample studies are needed to provide sufficient evidence for identifying the correlation between serum CAR combined with HCY/HDL-C and MVA. In addition, the data of CRP, ALB, HCY, and HDL-C were all obtained from a single blood sample before coronary angiography, and continuous dynamic detection has not been conducted. Whether CAR and HCY/HDL-C will change after coronary angiography deserves further attention and research.

## CONCLUSION

CAR and HCY/HDL-C, to a certain extent, respectively, reflect the inflammatory response and lipid metabolism abnormalities in patients and can better predict MVA. The combination of the two has higher diagnostic value, and it may become a potential, easy-to-measure, and economical parameter for the diagnosis of MVA.
